# Brief Contribution to the Elucidation of Syphilis

**Published:** 1890-10

**Authors:** John Hall

**Affiliations:** Victoria, B. C.


### BRIEF CONTRIBUTION TO THE ELUCIDATION OF SYPHILIS.

John Hall, M. D., Victoria, B. C.

The writer does not consider himself as much of a surgeon, or that he has had a large experience in the treatment of syphilitic disease, for it is well known that any noise made about such maladies effectually deters many from consulting, fearing, as they do, that they may be classed among them, consequently a large number of those suffering in this manner keep away, making our practice limited.

Still, the disease must be met and treated. Sometimes in its most virulent forms, demanding the ablest skill of our very best men to do this successfully. One poor fellow who fell under my care was taken down with a phagedenic chancre, of which he was so ashamed and ignorant as to hope that it might get well of itself, allowing it to progress so rapidly that when first seen the diseased organ was almost destroyed, requiring two eminent surgeons to patch as best they could the terrible consequences which had ensued, so that, while the above is an extreme case, the calling attention to our treatment of this disease is extremely appropriate.

Some time since, Ricord stated before his class "that he would not consent to have the smallest chancre on his organ for the largest fortune which could be left him as an inducement" (a remark which can be fully confirmed by his writings), but giving, as the leading physician in Europe at that time in this malady his inability to treat or cope with it, that the primary abrasion was as likely to be followed by secondary and tertiary symptoms, descending even to offspring in its baleful effects as if no treatment had been followed. The opinion of this great man may be taken as a fair experience of his school, sentiments which we need not enlarge upon. The question is, Can our homœopathic knowledge do better? We cannot doubt that there is existing great diversity of opinion and practice on this question even among us—some asserting that Hahnemann himself hardly knew what he was treating; that he was ignorant of the distinction between an indurated and a phagedenic chancre, classing both conditions alike, which our more elaborate researches in diagnosis have made plain, but whatever view we take, the *treatment* is still the problem. Leaving, then, gonorrhœa for a future consideration, we have this query:

Is syphilitic poisoning subject or amenable to homœopathic treatment so that the secondary and tertiary symptoms may be safely and fully removed in such degree that no after ill consequences can follow?

The writer, as before said, has had only a limited experience to draw from, but from what has been thus revealed he is inclined to think that the infection after a hard chancre, which may be two or three weeks in developing—that is, before any ulcer or outward sign appears whereby the poison may be recognized—may require to be imitated, so far by the appropriate medicine as requiring some time for its action before repetition, while a soft or phagedenic chancre, which usually reveals itself some two or three days after exposure, may require a more frequent repetition, an observation which has been confirmed to the author several times, especially since the higher potencies were used.

The object of this paper is to concentrate the knowledge of able men on this subject, enabling us more readily to find the true remedy for each case, which cannot be very difficult in the primary stages, as they do not greatly diverge, and then when found persistently to go ahead.

